# A new target for treating intervertebral disk degeneration: gut microbes

**DOI:** 10.3389/fmicb.2024.1452774

**Published:** 2024-11-29

**Authors:** Kaizhong Wang, Xiangyan Liu, Huagui Huang, Moran Suo, Jinzuo Wang, Xin Liu, Jing Zhang, Xin Chen, Zhonghai Li

**Affiliations:** ^1^Department of Orthopedics, First Affiliated Hospital of Dalian Medical University, Dalian, Liaoning, China; ^2^Key Laboratory of Molecular Mechanism for Repair and Remodeling of Orthopedic Diseases, Dalian, Liaoning, China; ^3^Musculoskeletal Research Laboratory, Department of Orthopedics & Traumatology, Faculty of Medicine, The Chinese University of Hong Kong, Hong Kong, China; ^4^Dalian Innovation Institute of Stem Cell and Precision Medicine, Dalian, Liaoning, China

**Keywords:** gut microbes, intervertebral disk degeneration, low back pain, spine, inflammatory, gut-disk axis

## Abstract

Intervertebral disk degeneration (IDD) is a common clinical spinal disease and one of the main causes of low back pain (LBP). Generally speaking, IDD is considered a natural degenerative process with age. However, with the deepening of research, people have discovered that IDD is not only related to age, but also has many factors that can induce and accelerate its progression. In addition, the pathogenesis of IDD remains unclear, resulting in limited traditional treatment methods that cannot effectively prevent and treat IDD. Conservative treatment may lead to patients’ dependence on drugs, and the pain relief effect is not obvious. Similarly, surgical treatment is highly invasive, with a longer recovery time and a higher recurrence rate. With the deepening of exploration, people have discovered that intestinal microorganisms are an important symbiotic microbial community in the human body and are closely related to the occurrence and development of various diseases. Changes in intestinal microorganisms and their metabolites may affect the body’s inflammatory response, immune regulation, and metabolic processes, thereby affecting the health of the intervertebral disk. In this context, the gut microbiota has received considerable attention as a potential target for delaying or treating IDD. This article first introduces the impact of gut microbes on common distal organs, and then focuses on three potential mechanisms by which gut microbes and their metabolites influence IDD. Finally, we also summarized the methods of delaying or treating IDD by interfering with intestinal microorganisms and their metabolites. Further understanding of the potential mechanisms between intestinal microorganisms and IDD will help to formulate reasonable IDD treatment strategies to achieve ideal therapeutic effects.

## 1 Introduction

LBP is a very common symptom worldwide. It occurs at various economic levels in countries and people of all ages, ranging from children to the elderly population, and lumbago is the main cause of global disability. As the population ages, LBP in our country has become a social public health problem, causing serious economic burdens on people’s lives and society (Yao et al., 2011; Buchbinder et al., 2018; Hartvigsen et al., 2018; Wu et al., 2020). The incidence of LBP caused by disk degeneration is 40%, and disk degeneration is considered the main cause of LBP (Yao et al., 2011). Although IDD has been intensively studied, the exact pathophysiology is poorly understood. Known etiologies of IDD include aging, cytoreduction, and altered ECM composition, which cause disrupted homeostasis, as well as changes in IDD function and structure (Vergroesen et al., 2015). Moreover, external factors such as smoking, trauma, obesity, nutritional and metabolic disorders, abnormal mechanical loading, environment and genetics can also lead to IDD through the induction of inflammation, apoptosis, proinflammatory cytokine secretion and autophagy (Risbud and Shapiro, 2014; Vo et al., 2016; Ohnishi et al., 2022).

Because the mechanism of disk degeneration is not clear, current IDD treatments can only relieve the patient’s symptoms with drugs or surgery. Conservative treatment may result in patient dependence on medications and inadequate pain relief. Similarly, surgical treatment is highly invasive and often requires a longer postoperative recovery time, as well as a high recurrence rate (Fan et al., 2023). This requires new treatment guidance. With the study of IDD pathogenesis and influencing factors, people have gradually found that gut microbes and many closely related organs, and use “axis” to represent the relationship between the intestinal tract, microorganisms and other organs, such as the “gut-bone axis” (Kohgo, 2001), “gut-brain axis” (Travagli and Rogers, 2001), and “gut-liver axis” (Zeuzem, 2000). Rajasekaran (Rajasekaran et al., 2020) and others found that there was microbial overlap between gut microbes and the intervertebral disk and that changes in gut microbes had a subtle impact on the degeneration of the intervertebral disk. The intestinal bacterial genera associated with IDD include *Family Rikenellaceae*, id.967, *Family Ruminococcaceae*, id.2050, *Genus Escherichia Shigella*, id.3504, *Genus Eubacterium coprostanoligenes group*, id.11375, *Genus Gordonibacter*, id.821, *Genus Lachnoclostridium*, id.11308, *Genus Oscillospira*, id.2064, *Phylum Bacteroidetes*, id.905, etc (Geng et al., 2023). The concept of the “gut-intervertebral disk axis” was also proposed in Li et al. (2022). This review summarizes the effects of gut microbes on disk degeneration and summarizes the progress of several studies.

## 2 Structure of the IVD

The IVD is an avascular tissue that receives only small arteries that supply the outermost fibers of the AF and is composed of fibrous tissue and cartilage (Boxberger et al., 2009; Mohd Isa et al., 2022). Its main function is to transfer compressible loads between vertebrae while providing flexibility (Dowdell et al., 2017). Normal anatomy underlies the physiological function of the IVD (Lin et al., 2023). The intervertebral disk (IVD) is a strong, flexible structure found between neighboring vertebrae and consists of the nucleus pulposus (NP), annulus fibrosus (AF), and cartilage endplate (CEP) (Colombini et al., 2008).

The AF is a ring of fibers composed of highly organized 15 to 25 concentric collagen fiber layers called sheets that surround the NP. Each sheet is formed from tough collagen fibers tilted approximately 30° from one vertebra to another (Mohd Isa et al., 2022). The fibers of adjacent sheets cross each other in opposite directions at an angle of more than 60. This arrangement allows for limited rotation and bending between adjacent vertebrae and enables the IVD to withstand the circumferential load (Molladavoodi et al., 2020; Mohd Isa et al., 2022). The NP is the core of the IVD. Between 70% and 90% of its dry weight is composed of water, with 35–65% proteoglycan, 5–20% thin type II collagen fibers, and the remaining noncollagen and elastin. Proteoglycan retains the high water content of the NP and is hydraulically distributed to the ECM to reduce the stress of the IVD (Lv et al., 2014). CEP is a hyaline cartilage buffer composed of round cells located between the vertebral endplate and NP. It acts as a physical barrier and a conduit for supplying nutrients to the intervertebral disk (Moon et al., 2013). The CEP consists of approximately 60% water, and the main dry weight components are type II. collagen and proteoglycans (Newell et al., 2017). Morphologically, CEP contains elongated cells parallel to the IVD that are aligned with collagen fibers, and the cells produce a collagen-rich transmembrane matrix and a proteoglycan-rich regional matrix (Lakstins et al., 2021).

## 3 Factors influencing IDD

**FIGURE 1:**
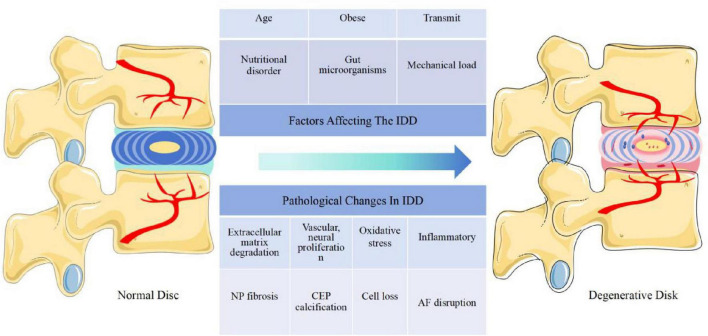
The IVD is affected by many factors (age, mechanical load, obesity, gut microorganisms, nutritional disorders, transmission, nutritional disorders, etc.) and undergoes a series of pathological changes (cell loss, inflammation, extracellular matrix degradation, NP fibrosis, vascular, neural proliferation, CEP calcification, AF disruption, oxidative stress, etc.) leading to IDD. Abbreviations: IVD, intervertebral disk; IDD, intervertebral disk degeneration; NP, nucleus pulposus; CEP, cartilage endplate; AF, annulus fibrosus.

The induction and progression of IDD is a complex process that is usually affected by multiple factors such as age, genetics, non-physiological mechanical loading, obesity, and nutritional disorders. Under the combined effect of these factors, many pathological changes occur, such as inflammation, ECM degradation, oxidative stress, mitochondrial dysfunction, IVD cell destruction, loss of physiological cell function, ECM metabolic disorder, NP fibrosis, AF disintegration and CEP calcification. This ultimately leads to the reduction of IVD mechanical properties, IVD height, and NP size, leading to the emergence and development of IDD.(Guo et al., 2022; Xu et al., 2023; Figure 1).

Research has indicated that cellular senescence increases with age, featuring permanent cessation of the cell cycle and a senescence-linked secretory phenotype (SASP). Senescent cells (SCs) that release SASP products produce numerous substances, including inflammatory cytokines, growth regulators, chemokines, angiogenic factors, and matrix metalloproteinases (MMPs)(Gorgoulis et al., 2019; Patil et al., 2019; Xu et al., 2023). The expression and activity of MMP subgroups in IVD are significantly enhanced. Due to their important role in ECM deterioration and remodeling, the expression and activity of MMP subgroups in IVD are significantly enhanced, leading to the destruction of ECM balance and induction of IDD (Zou X. et al., 2023). Moreover, the SASP produced by the SCs in the IVD can disrupt the metabolic balance in the ECM, leading to an imbalance of the IVD *in vitro* and *in vivo* and inducing the development of IDD (Wu et al., 2022; Xu et al., 2023). In addition, mitochondrial dysfunction and oxidative stress often occur in aging organisms and can cause mitochondrial autophagy and NP cell apoptosis, thus leading to IDD (Xu et al., 2019).

In addition to age, researchers have found a relationship between IDD and genetics, and many of the genes were identified as having a single nucleotide polymorphism (SNP) that affects the risk of developing IDD (Martirosyan et al., 2016; Dickinson and Bannasch, 2020). Prolonged compression, strain, and acute nonphysiological loading all lead to a series of changes in the IVD, such as tissue damage, reduced vascular sprouting, reduced cell activity, inflammatory factor release, and ECM degradation, ultimately leading to IDD (Zhan et al., 2020; Zhan et al., 2021; Zhou Z. et al., 2021). Because the IVD lacks a blood supply, the IVD is a hypoxic, low-nutrient acidic microenvironment (McDonnell et al., 2023). In this environment, it leads to cell death, decreased metabolic activity, and activation of autophagy, for example, in AF cells (Yurube et al., 2019). In addition, these factors can also lead to the activation and enhancement of autophagic activity within the IVD, especially in NP cells, which eventually leads to the development and progression of IDD (Kritschil et al., 2022). In addition, a two-sample Mendelian randomization study revealed that IDD is closely linked to body mass index (BMI), and when a person is obese, the risk of IDD greatly increases (Zhou J. et al., 2021). The poor blood supply of the IVD and the poor ability to repair itself, when affected by the above pathological or physiological factors, can induce IDD or accelerate IDD development.

## 4 The role of the GM in the human body

**FIGURE 2:**
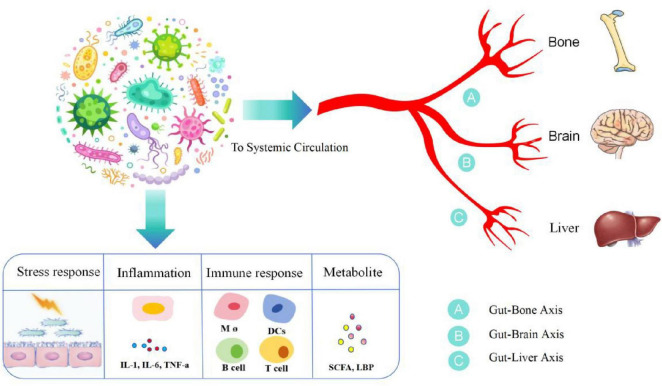
Gut microorganisms and various distal organs constitute a pathway of influence, which we call axes, such as the gut-bone axis, the gut-brain axis, and the gut-hepatic axis. Gut microorganisms, under the influence of stress response, inflammation, immunity, etc., produce a variety of inflammatory mediators, such as IL-1, IL-6, and tumor necrosis factor, and a variety of immune cells, such as B cells, T cells, and monocytes, as well as the metabolites SCFA and LPS, which arrive at distal organs through the blood circulation and are involved in disease progression. IL-1, interleukin 1; IL-6, interleukin 6; SCFA, short-chain fatty acid; LPS, lipopolysaccharide.

Microorganisms inhabit all the surfaces of the human body, but large numbers of them live in the gastrointestinal tract. The human gut harbors approximately one thousand microbial species that form a complex ecological community called the gut microbiota (Lagier et al., 2016). The gut microbiome has recently been classified as a “vital organ”. It not only plays an important role in maintaining the homeostasis of the intestinal ecosystem and intestinal nutrient absorption, but also interacts with the host immune system, enhances immune response, defends against pathogens, and helps maintain the intestinal barrier function to prevent harmful substances from entering the blood. Participate in the metabolism of fats, sugars, and proteins. Certain intestinal microorganisms are able to synthesize vitamins (such as vitamin K and some B vitamins), which are essential for maintaining health. The composition of each person’s intestinal microorganisms is unique and is influenced by multiple factors such as genetics, diet, and environment. This difference may affect health status and susceptibility to disease (Gomes et al., 2018; Yuan et al., 2021). Communicate with other organs by forming interconnected “axis”-like pathways Any change in the microbial community not only leads to gut-related problems but also affects other organ-related diseases, although the actual mechanisms of interaction between the gut and organs are not yet fully understood (Afzaal et al., 2022). Typical progressive studies of intestinal microgrowth on other organs include studies on the intestinal-bone axis, intestinal-brain axis, and intestinal-liver axis (Figure 2 and Table 1).

**TABLE 1:** Studies related to the effects of gut microbes on bone, brain and liver.

Way	References	Journal	Results
Gut-bone axis	Bora et al., 2018	Front Immunol	The Gut Microbiota Regulates Endocrine Vitamin D Metabolism through Fibroblast Growth Factor
	Zaiss et al., 2019	Clin Invest	The Gut Microbiota is a key regulator of bone health that affects postnatal skeletal development and skeletal involution
	Lin et al., 2023	Nat Commun	The Gut microbiota impacts bone via Bacteroides vulgatus-valeric acid-related pathways
	Wu et al., 2022	Agric Food Chem	The Quercetin may improve bone loss by restoring gut microbiota
Gut-brain axis	Zhu et al., 2020	Neuroinflammation	The dynamic changes in the gut microbiota can alter brain physiology and behavior
	Zou et al., 2022	Front Immunol	The Gut Microbiota have an impact on brain-related diseases through the gut-brain axis pathway
	Zhu Z. et al., 2022	Front Pharmacol	The Side effects of the gut-brain axis in lipid metabolism
Gut-liver axis	Minemura and Shimizu, 2015	Gastroenterol	The effect of gut microbes on liver disease through the enteric liver axis
	Hrncir et al., 2019	Lab Anim	The role of gut microbiota in intestinal and liver diseases
	Chassaing et al., 2014	Hepatology	The important influence of gut microbes in liver disease

### 4.1 Gut-bone axis

The gut microbiota plays a key role in bone formation and development (Seely et al., 2021). Calcium is a key nutrient essential for bone health, and calcium deficiency can cause osteopenia in individuals with osteoporosis (Chen et al., 2022). Gut microbes play an important role in the homeostasis of bone (Behera et al., 2020). Raveschot et al. reported that probiotic supplements (Lactobacillus strains) are beneficial for the transport and uptake of calcium in mice. Probiotics are defined as “living microorganisms”, which proves the importance of calcium absorption by microorganisms for bone development (Holick, 2007; Behera et al., 2020). Vitamin D has a very significant impact on the absorption of calcium and phosphorus and on bone metabolism in the human body, and Zu et al. reported that an imbalance of the intestinal flora limits the production of vitamin D (Holick, 2007; Zuo et al., 2019). Bora et al. (2018) used mice with microorganisms and not microbiota to observe the ability of vitamin D to regulate calcium metabolism and found that the levels of 1,25-dihydrooxygen vitamin D and calcium were restored in microbial mice. In addition, Zaiss et al. (2019) A dynamic study revealed that SCFAs, one of the major metabolites of gut microorganisms, can blunt osteoclast-mediated inhibition of histone deacetylase (HDAC) activity; thus, osteoclast differentiation prevents the prevention of bone loss, and it was also found that dietary supplementation with SCFAs, which generate oligosaccharides, can increase bone mineral density (BMD), thus affecting bone development. Lin et al. (2023) showed that ovariectomized mice fed B. vulgatus exhibited increased bone resorption and poor bone microstructure, whereas mice fed serum valeric acid (VA) showed decreased bone resorption and improved bone microstructure. VA inhibits RELA protein production (proinflammatory) and enhances IL-10 mRNA expression (anti-inflammatory), resulting in the inhibition of osteoclast-like cell maturation and enhanced osteoblast maturation *in vitro*.

Furthermore, Li et al. (2023) in an ovariectomy (OVX) mouse model, OVX induced a surprising disruption of the microbial community, leading to intestinal leakage, intestinal barrier dysfunction and the aggravation of postmenopausal osteoporosis (PMO). Additionally, lipopolysaccharide (LPS)-triggered inflammatory cytokines released from the intestine to the bone marrow were found to be associated with bone loss in ovariectomized (OVX) mice. Long-term dietary isisquercetin was used. The microbial community and gut barrier function improved in OVX mice. Thus, the inhibition of the NF-KB signaling pathway significantly improved bone loss and the host inflammatory status, and isoquercetin treatment dose-dependently inhibited the inflammation induced by LPS. In addition, it partially promoted the proliferation and differentiation of osteoblasts. These advances were all conducted through the gut-bone axis while reflecting the influence of gut microorganisms on bone physiopathology through multiple pathways in the intestinal-bone axis.

### 4.2 Gut-brain axis

Mounting evidence indicates that fluctuations in the gut microbiome can affect brain function and behavior. It was once believed that cognition was solely controlled by the central nervous system. However, it is now becoming clear that many nonneurological factors, including gut-resident bacteria of the gastrointestinal tract, regulate and influence cognitive dysfunction and processes as well as neurodegenerative and cerebrovascular diseases (Zhu et al., 2020).

In the study of Zou et al. cerebrovascular disease (CeVD) has high morbidity, mortality, and disability, posing a serious threat to human health (Zou et al., 2022). Gut bacteria significantly affect the onset, progression, and prognosis of CeVD. The gut microbiota plays a key role in gut–brain interactions, and the gut–brain axis is critical for CeVD communication. Olanzapine (OLZ) is a representative atypical antipsychotic (AAPC) prone to cause weight gain, obesity, hyperglycemia and dyslipidemia after long-term use (Visconti et al., 2019). Zhu Z. et al. (2022) studied the side effects of olanzapine-induced lipid metabolism disorders and reported that olanzapine increased the Firmicutes/Bacteroidetes (F/B) ratio and reduced the abundance of serotonin (5-HAT). SCFAS, one of the main metabolites of intestinal microbes, was proven to promote the secretion of 5-HT by stimulating receptors on vagal nerve 3 vagal nerve endings via 5-HT, thus regulating the body’s lipid metabolism (Browning, 2015). The vagus nerve is a physiological link between gut microbes and the central nervous system and regulates the host appetite by transmitting gut hormone signals secreted by the gut (Cork, 2018). After receiving signals that involve serotonin from the gut, the brain controls hunger by producing the neuropeptide Y/rat-related peptide (NPY/AgRP) in the hypothalamus. By acting as appetite stimulants, NPY and AgRP can prompt eating and decrease the amount of energy used (Schwartz et al., 2000; Savontaus et al., 2002). This study also demonstrated a link between gut microbes and the brain.

In addition, Zhu R. et al. (2023) reported that the intestinal microflora in Alzheimer’s disease (AD) patients decreased, the intestinal flora of AD mice improved in response to probiotics, and the AD symptoms improved. Zhu H. et al. (2022) reported that perinatal transmission of probiotic Bifidobacterium strains prevented emotional and gastrointestinal motility disorders caused by stress early in life. Zou L. et al. (2023) reported that a diet high in cholesterol also promoted anxiety and depression in mice by affecting gut microbes. According to the above studies, there is an interconnection between the intestinal microbiome and the brain, and many related mechanisms of disease occurrence and progression are achieved through the gut-brain axis.

### 4.3 Gut-liver axis

Marshall introduced the idea of the “liver-gut” axis in 1998, sparking increased interest in the connection between the gut and liver. The liver is greatly impacted by changes in the intestinal flora as intestinal bacteria or their byproducts travel to the liver through the portal vein. Understanding the liver-gut axis is crucial for understanding the pathophysiology of various liver diseases, such as nonalcoholic fatty liver disease and hepatic encephalopathy (Minemura and Shimizu, 2015; Hrncir et al., 2019; Guo et al., 2022; Zucoloto et al., 2023).

Through this research, Chassaing et al. (2014) found that changes in the gut microbiota composition or altered barrier function can lead to the activation of Toll-like and Nod-like receptors in the innate immune system by gut microbial products. TLR/NLR activation can drive proinflammatory gene expression, thereby promoting liver disease. Changes in people’s dietary habits include increased intestinal permeability, increased serum endotoxin levels, and moderate increases in the levels of proinflammatory cytokines associated with various aspects of metabolic syndrome (including NAFLD) (Pendyala et al., 2012). The alteration of the intestinal microbiota and permeability due to alcohol can result in heightened activation of the liver TLR/NLR, thus leading to the emergence of liver disease (Lu et al., 2011). Additionally, lipopolysaccharide, which is a toxic substance originating from gram-negative bacteria found in the intestines, contributes to the progression of liver damage by stimulating liver cells to generate TNF. TNF then travels through the bloodstream and enhances the permeability of the blood-brain barrier, ultimately causing hepatic encephalopathy (Ma et al., 2004).

Many studies have shown that gut microbes participate in the progression of HBV through their metabolites and influence autoimmunity and can also be used as markers of the prognosis of patients with HBV (Xu et al., 2012; Yang et al., 2018; Sun et al., 2021; Wang et al., 2021). People also use the interaction between intestinal microbes and the liver to intervene in and treat liver diseases. For example, Guo et al. (2022) others have used traditional Chinese medicine to act on the intestinal liver axis to intervene in the treatment of nonalcoholic fatty disease (NAFLD).

In addition, Shahbazi et al. (2023) reported that advanced liver disease and cirrhosis alter the composition of the gut microbiota and that altering the microbiota produces different metabolites, damages the gut barrier, leads to bacterial translocation, and alters bacterial metabolites and products (i.e., DNA, LPS, etc.). By triggering the innate immune system, which initiates the production of proinflammatory cytokines, systemic inflammation activates sinusoidal blight cells, leading to hepatocellular damage, hepatocellular damage with gut-derived products and metabolites compromising the integrity of the blood–brain barrier (BBB). Restoration of the gut flora improves cognitive dysfunction in hepatic encephalopathy (Zhu J. et al., 2023). All of the above studies reflect the interaction between the gut microbiota and liver disease and the progress of research on the gut-liver axis.

## 5 The GM and IDD

Researchers have never stopped studying the effect of microorganisms on disk degeneration, the most representative being the effect of Propionibacterium acnes on disk degeneration (Table 2). Chen et al. (2016) observed changes in MRI and histology by injecting Propionibacterium acnes, *Staphylococcus aureus* (*S. aureus*) and rabbits. The control showed that the disk caused changes and intervertebral discitis caused by *S. aureus*, and the pathological changes caused by *Propionibacterium acnes* (*P. acnes*)were considered to be Modic-I changes and disk degeneration; however, Ahmed-Yahia et al. (2019) However, whether acnes cause Modic-I changes has not been determined through clinical experiments. Yuan et al. (2017) reported that histological evidence also supported a link between *P. acnes* and disk degeneration. Clinical trials such as those by Magno da Rocha et al. (2023) are also exploring the link between the two. Research has revealed that the influence of *P. acnes* on disk degeneration causes pyroptosis of the nucleus pulposus via ROS-NLRP3 and exacerbates disk deterioration (Tang et al., 2021). In addition, *P. acnes* promotes cell apoptosis in the nucleus pulposus through the TLR 2/JNK/mitochondria-mediated pathway and induces intervertebral disk degeneration (Lin et al., 2018). In addition, Lan et al. (2022) found that different systemic types of acne caused different patterns of disk degeneration. With the continuous exploration of the influence of microorganisms on disk degeneration, whether the intestinal microbiota is involved in disk degeneration has become a hot topic in related research.

**TABLE 2:** Recent correlative studies on the effects of *P. acnes* on IDD.

References	Journal	Results
Chen et al., 2016	BioMed Research International	*P. acnes* causes Modic-1 changes and disk degeneration
Yuan et al., 2017	BioMed Research International	*P. acnes* was absolutely confirmed to be present in some nonpyogenic degenerated intervertebral disks, at a prevalence of approximately 21.05%.
Lin et al., 2018	Emerging Microbes & Infections	*P. acnes*-induced apoptosis of NPCs via the TLR2/JNK pathway is likely responsible for the pathology of IDD.
Ahmed-Yahia et al., 2019	PLOS ONE	*P. acnes* is present in both the IDD and the IVD
Tang et al., 2021	Oxidative Medicine and Cellular Longevity	*P. acnes* induces NPC pyroptosis via the ROS-NLRP3 signaling pathway, and the pyroptotic NPCs cause an IDD cascade.
Lan et al., 2022	PLOS ONE	*P. acnes* is widespread in herniated disk tissues.
Magno da Rocha et al., 2023	PLOS ONE	*P. acnes* causing IDD and its association with LBP.

*P. acnes*, *Propionibacterium acnes*; NPCs, nucleus pulposus cells; TLR2, Toll-like receptor 2; JNK, c-Jun N-terminal kinase; IVD, intervertebral disk; IDD, intervertebral disk degeneration; LBP, low back pain; IDD, intervertebral disk degeneration.

**FIGURE 3:**
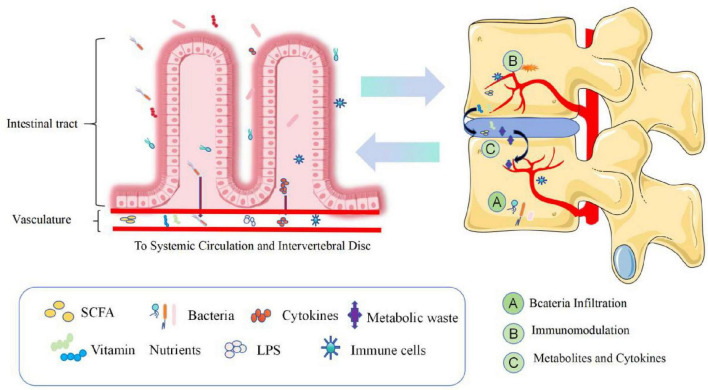
Dysregulation of intestinal homeostasis, intestinal bacteria and the production of metabolic wastes, various nutrients and immune cells, etc., enter the blood circulation and pass through **(A)** Bacteria Infiltration, **(B)** Injury-Induced Inflammation, **(C)** Metabolites and cytokines. Three pathways are involved in the process of disk degeneration. The intervertebral disk constitutes the intestinal disk axis. SCFAs, short-chain fatty acids; LPS, lipopolysaccharide.

Dysbiosis of the gut microbiota results in the abnormal production of several metabolites, signaling molecules, and immune cells that may affect the musculoskeletal system (Ratna et al., 2023). Li et al. (2022) described three potential mechanisms by which the GM induces IVD and leads to LBP (Figure 3 and Table 3). In the first pathway, bacteria cross the intestinal epithelial barrier and enter the IVD. In a low-acid hypoxic environment, microbes can colonize and multiply (McDonnell et al., 2023). When the intestinal microbial system is disturbed or intestinal inflammation occurs, the intestinal epithelial barrier is destroyed, the permeability of the intestine increases, and more intestinal bacteria pass through the intestinal epithelial barrier (Thevaranjan et al., 2018). Although most bacteria are eliminated by the immune system, a few escape the immune system, reaching bacteria near the IVD or entering the IVD by releasing proinflammatory factors (such as IL-6 and TNF-α) to recruit more inflammatory cells (such as T cells, B cells, dendritic cells and macrophages) (Schirmer et al., 2016) while stimulating IVD cells to secrete inflammatory cytokines, such as IL-1α/β, IL-17, TNF-α, and IL-6 (Risbud and Shapiro, 2014). Moreover, these cytokines destroy the IVD extracellular matrix (ECM) by promoting the degradation of aggrecan and collagen. A series of chain reactions initiated by these cytokines leads to the production of chemokines that further impair the ECM (Hampton and Chtanova, 2019). Furthermore, the release of inflammatory molecules secreted by the damaged IVD and the activation of macrophages, T and B cells, mast cells, and neutrophils further amplify the inflammatory cascade, which leads to IDD. Bacterial translocation into the IVD can also lead to the activation of these immune cells through the release of lipopolysaccharide (LPS) and cause persistent pain.

**TABLE 3:** Three potential pathways by which gut microbes may influence IDD.

Pathways	Mechanism of action
Bacteria Infiltration	Bacterial translocation across the gut epithelial barrier and into the IVD, when the intestinal barrier is adversely affected.
Immunomodulation	Dysbiosis of intestinal flora, bacterial contact with the mucosal immune system, promotes activation of the immune system and the release of large amounts of pro-inflammatory factors, which can accumulate toward and affect IVD.
Metabolites and Cytokines	Intestinal bacteria affect the absorption of nutrients by the intestinal epithelium and produce metabolites and cytokines that diffuse into and affect the IVD.

IVD, intervertebral disk degeneration; IDD, intervertebral disk degeneration.

The second pathway involves systemic and mucosal immunomodulation. Excessive contact between intestinal bacteria and the intestinal mucosa leads to the activation of the immune system and increases the number of immune cells that release competent proinflammatory factors such as IL-6 and TNF-α into the blood to regulate bone metabolism. These inflammatory cytokines and activated immune cells can migrate near the IVD. The infiltration of these products can cause long-range inflammation that causes IVD, and products such as SCFAS can affect the development of bone (Mortensen and Clausen, 1996; Zaiss et al., 2019).

The third path involves balancing nutrient absorption and the formation of intestinal epithelial metabolites and their diffusion into the IVD. In cirrhosis, diabetes, obesity, neurological diseases, and intestinal flora disorders, under the influence of these factors of intestinal toxins, inflammatory cytokines, microorganisms and microbial metabolites interfere with the production of mucin by cup cells and intestinal barrier damage, causing intestinal bacterial translocation and the transfer of toxic metabolites such as endotoxin/LPS, SCFAs/D-lactic acid and inflammatory factors (Sun et al., 2021; Li et al., 2022; Anachad et al., 2023). These inflammatory factors and metabolites can cause remote inflammation of the IVD, and SCFAs can promote the differentiation of primary CD4++ cells to Tregs. Tregs preferentially stay on the endosteal surface, promote osteoblast differentiation, and inhibit the formation of osteoclasts; at the same time, they are necessary for parathyroid hormone stimulation (parathyroid hormone, pth stimulation) and bone formation (Fujisaki et al., 2011; Qiu et al., 2015; Yu et al., 2018). In addition, SCFAs are removed by the activation of G protein-coupled receptors (GPCRs), or the inhibition of HDACs has a direct effect on bone resorption or osteoclast formation. Calcium deposition in the disk and the expression of extracellular calcium-sensitive receptors (extracellular calcium-sensing receptors, CaSR) are closely associated with GPCRs in degenerative disks, suggesting that the diffusion of intestinal-derived SCFAs to the IVD can lead to calcification and IDD (Kotterman et al., 2015; Li et al., 2022). Under the combined action of the above factors, the hypooxygen environment of infiltrating IVDs with inflammation is disrupted, and the ECM is destroyed by the degradation of cytokines, leading to IDD-related damage to the IVD (Risbud and Shapiro, 2014; Hampton and Chtanova, 2019).

As we all know, vitamin D plays an important role in human bone health (Bouillon et al., 2019). All cells that make up the bones (chondrocytes, osteoblasts and osteoclasts) contain vitamin D receptors and the enzyme CYP27B1, which are required for the production of the active metabolite of vitamin D, 1,25-dihydroxyvitamin D (Bikle, 2012). Intestinal microorganisms can affect the metabolism of vitamin D (such as Carnosaurus maltiferus), which in turn affects the health of bones (including intervertebral disks) (Li et al., 2023). In addition, it has been suggested that environmental factors such as pollution, diet, and lifestyle may play a crucial role in autoimmune diseases in conjunction with genetic traits (Murdaca et al., 2021). In particular, vitamin D has been negatively associated with the development of several autoimmune diseases. Murdaca and Gangemi (2022) found that vitamin D plays an important role in calcium homeostasis and bone metabolism, possesses anti-inflammatory and antioxidant properties, and acts on both innate and adaptive immunity. In the human body, gut microbes and vitamin D interact in many different ways to profoundly affect the immune system. Vitamin D is a key mediator in the interconnection between gut microbes and the immune system. It follows that gut microbes can influence their immune system by interfering with the metabolism of microbial vitamin D and thereby (Murdaca et al., 2021). This leads to a series of responses such as oxidative stress and inflammation, which further induce or accelerate IDD.

## 6 Current research status of GM and IDD

To verify the relationship between intestinal microorganisms and intervertebral disk degeneration. Whether it is expected to become a new target for intervention in IDD. The researchers conducted a series of studies (Table 4).

**TABLE 4:** Research progress on gut microorganisms and IDD.

References	Journal	Results
Yao et al., 2023	Orthop Surg	Intestinal flora has an impact on the progression of intervertebral disk degeneration, and FMT can be used as a promising target to improve IDD.
Rajasekaran et al., 2023	Spine J	The presence of bacteria may not just be contamination, but may be colonization and may play a role in inflammation in IDD.
Geng et al., 2023	J Orthop Surg Res	There is a causal relationship between intestinal flora and IDD, and nine bacterial genera that have the strongest causal relationship with IDD have been detected.
Wang et al., 2024	Phytomedicine	By regulating intestinal flora, it can reduce the inflammatory response, inhibit ECM degradation, restore IVD height and water content, and have obvious therapeutic effects on IDD.
Fang et al., 2024	Eur Spine J	Some intestinal microorganisms and their metabolic pathways are causally related to IDD, LBP and sciatica and can be used as potential intervention targets.
Cheng et al., 2024	J Orthop Surg Res	Gut microorganisms are closely related to IDD and play an important role in the process of IDD.

IVD, intervertebral disk degeneration; IDD, intervertebral disk degeneration; FMT, fecal microbiota transplant.

Mendelian randomization (MR) is a statistical method used to estimate the causal effect of exposure factors (e.g., lifestyle or biomarkers) on outcome variables (e.g., disease or health outcomes) and is often used to verify the causal effect of both factors. Geng et al. (2023) used the inverse variance weighting (IVW) method as the main MR (two-way two-sample Mendelian randomization study) analysis method. The weighted median, MR-Egger regression, weighted mode and simple mode were used as supplements. Level pleiotropy (MR-outliers PRESSO) and MR-Egger regression. Cochran’s Q test was used to assess heterogeneity, and reverse MR analysis was used to assess potential reverse causality. Two-way two-sample Mendelian randomization analysis of 211 gut microbial taxa and IDD revealed eight nominal causalities and strong correlations, which further supported the concept of the disk axis. Fang et al. also used Mendelian randomization studies to verify the causal relationship between gut microbiota and IDD (Fang et al., 2024).

In one study, 6 disks (3 controls and 3 cases) were selected for mass spectrometry identification. After excluding metabolites related to humans, drugs, and food, 39 bacterial-specific metabolites were isolated. Nine metabolites were found to have significant fold changes > 1.0, including (S)-14-methylhexadecanoic acid related to *P. acnes*, 9-OxoODE and 13-OxoODE related to intestinal flora, Vibrio (siderophores), tuberculin and isotuberculol, virulence factors of Mycobacterium tuberculosis. This also provides evidence for the involvement of intestinal microorganisms in IDD (Rajasekaran et al., 2023).

Using 2-month-old male Sprague–Dawley rats, Yao et al. (2023) established an experimental model of IDD. FMT (fecal microbiota transplantation) was performed by gastric gavage of IDD rats with fecal bacterial solution. After surgery, rat serum, feces and disk tissue were collected for 2 months. TNF-α, IL-1β, IL-6, matrix metalloproteinase (MMP)-3, MMP-13, and collagen II. The levels of aggrecan were assessed using enzyme-linked immunosorbent assay, immunohistochemistry, real-time polymerase chain reaction, or protein blotting. The pathology of the disk tissue was also examined using hematoxylin and eosin (HE) and red O-fast green staining. The intestinal microbiota in rat feces was also examined using 16S rRNA gene sequencing. In the IDD group, the rats in the IDD group had severely impaired disk tissue, disordered cell arrangement, uneven pupanin coloring, and increased expression of TNF-α, IL-1β, IL-6, MMP-3, MMP-13, NLRP3 and Caspase-1, while collagen II and aggrecan levels were decreased. FMT reversed the effects of IDD on these factors and alleviated damage to cartilage tissue. FMT increased the gut microbiota diversity and microbial abundance in IDD-treated rats. This finding also provides further evidence for the effect of the gut microbiota on IDD. Cheng et al. also used 16S rRNA gene sequencing to verify the impact of intestinal microorganisms on IDD and the relationship between the two in an IDD model established in rabbits (Cheng et al., 2024).

In the latest study, Wang et al. established a rat IDD puncture model and treated it with Sanbi decoction (SBD) tube feeding at different concentrations. After 4 weeks, clean feces, serum, liver, kidney and intervertebral disk (IVD) were collected and tested using the same method as Yao et al. The results show that SBD can extensively regulate intestinal flora and serum metabolic homeostasis, reduce inflammatory responses, inhibit ECM degradation, restore IVD height and water content, and has a significant therapeutic effect on IDD. This provides a basis for intestinal microorganisms to become a new target for the treatment of IDD, and further proves the connection between intestinal microorganisms and IDD (Wang et al., 2024).

## 7 GM regulation therapy for IDD

Traditional treatments such as medication and surgery are commonly used for IDD, and the global prevalence of this condition highlights the critical necessity for impactful therapeutic solutions that can help ease symptoms and delay the progression of IDD. In recent years, with continuous research and exploration, many promising influences as well as therapeutic approaches to improve and treat IDD have been identified (Samanta et al., 2023). Gut microbes are promising targets for therapeutic strategies because they can be modified by lifestyle changes, such as dietary interventions, sleep and exercise, fecal transplantation, and future microbiome-targeted therapy (Konturek et al., 2015; Morimoto et al., 2023).

### 7.1 Lifestyle changes

A healthy lifestyle can improve gut microbial abundance and species (increasing “good” bacteria) and may improve gut flora disorders (Li et al., 2016). Improving intestinal dysbiosis can reduce immune and inflammatory responses, improve the environment of the IVD, delay the process of IDD, and improve bone quality by relieving pain through short-chain fatty acids or neurotransmitters (Hampton and Chtanova, 2019; Zaiss et al., 2019). A high-fat diet can cause unhealthy obesity and increase cholesterol in the body. Morimoto et al. (2023) reported that cholesterol can induce cell pyroptosis and matrix degradation through mSREBP1-driven intervertebral disk degeneration. Prebiotics, such as fiber and oligosaccharides, are indigestible substances found in food. They promote the growth and function of helpful bacteria in the gut, leading to positive effects on health by assisting in the production of SCFAs, influencing cell development, hormone release, and inflammation control, and providing advantages to the host. It also plays an important role in maintaining healthy gut microbial homeostasis by promoting epithelial barrier function and reducing dysbiosis, stimulating the production of antimicrobial substances and immunoglobulin, and inhibiting the production of bacterial toxins, thus promoting the host immune response and anti-inflammatory pathways (Li et al., 2016; Zhang et al., 2023). In conclusion, maintaining a healthy lifestyle can maintain or improve intestinal homeostasis, and the maintenance of gut microbes reduces the range of its effects on disk degeneration.

### 7.2 FMT

FMT refers to the transplantation of processed healthy populations of fecal flora to patients. FMT can rebuild the intestinal flora, regulate intestinal flora imbalance, and rebuild the normal function of the intestinal ecosystem, with curative effects, fewer side effects, a short course, and a low recurrence rate. FMT has been used to treat gastrointestinal diseases, including constipation, inflammatory bowel disease, Parkinson’s disease, and multiple sclerosis (Wang et al., 2022; Zhang et al., 2022). Yao et al. (2023) in other experiments, FMT was used to verify the relationship between the gut microbiota and IDD, and FMT improved the effect of intestinal dysbiosis on IDD through FMT. FMT, or fecal microbiota transplantation, refers to the transplantation of treated fecal flora from healthy people into patients to rebuild the intestinal flora, regulate the imbalance of intestinal flora, and reestablish the normal function of intestinal ecosystems. Fecal microbiota transplants, which have the advantages of good efficacy, few side effects, a short course of treatment, and a low recurrence rate, have been used to treat gastrointestinal disorders, including constipation, inflammatory bowel disease (IBD), parkinsonism, and multiple sclerosis (Zhu H. et al., 2022; Zhu J. et al., 2023). Zhan et al. (2021) constructed an IDD model using rats, verified the relationship between the gut microbiota and IDD using FMT, and demonstrated that FMT ameliorated the effects of gut flora dysbiosis on IDD. Cheng et al. constructed an IDD model using rabbits, and the results showed that IDD altered the intestinal microbiota and fecal metabolites in the rabbit model and that multiple metabolites were elevated in the IDD model in association with a variety of gut bacteria. These results provide a direction and theoretical basis for the clinical application of FMT to modulate the gut flora in the treatment of IDD-induced low back pain (Cheng et al., 2024). FMT may be a promising target for improving IDD and palliative care and may become a promising target for improving IDD and palliative treatment.

When gut microorganisms are disrupted in the intestinal environment due to diseases of other organs or systems and the intestinal flora is dysregulated, all of these conditions can induce the development of IDD or accelerate the process of IDD. Conversely, the gut flora can be restored by therapeutic interventions in the gut-disk axis to prevent the onset and progression of IDD (Yao et al., 2023; Wang et al., 2024).

## 8 Conclusion and perspective

Ever since Rajasekaran discovered that intervertebral disks and gut microbes share bacterial genera, scientists have been exploring the relationship between the two. In animal models, it has been confirmed that intestinal microorganisms and their metabolites are involved in the formation and progression of IDD. However, the research on the involvement of intestinal microorganisms and their metabolites in IDD is still in the preliminary exploration stage. Intestinal microorganisms, as well as calcium, phosphorus, short-chain fatty acids SCFA, immune factors, etc. The imbalance of these intestinal flora and substances produced by metabolism may lead to changes in the body’s immunity and inflammation, and further cascade reactions may occur, affecting the normal function of IVD. Structure and function. Therefore, an in-depth understanding of the potential mechanisms by which intestinal microbes participate in IDD, as well as exploring strategies to intervene in the impact of intestinal microorganisms on IDD, will be beneficial to the subsequent treatment of IDD.

Gut microbial therapies targeting IDD have been partially investigated in preclinical studies with success. But there are also shortcomings. Although most preclinical studies are able to reflect the relationship between the two, as well as the changes in IDD and its mechanisms when changing the intestinal microbiota, heterogeneity exists between organisms. IDD is a gradual development process. Only changes in the intestinal flora of experimental animals with severe IDD and normal controls were observed. The sample size is small and the results are not generalizable. In future studies, researchers need to increase the sample size, dynamically observe the changes in intestinal flora from mild to severe IDD, and conduct clinical studies in a timely manner to explore whether the two have the same relationship in the human body. To gain a deeper understanding of the mechanism by which intestinal microorganisms participate in IDD, as well as the changes in intestinal microorganisms during the process of IDD. It will provide innovative ideas and strategies for the treatment of IDD and lay the foundation for intestinal microorganisms to become a new target for delaying or treating IDD.
